# The association between the gut microbiome and antituberculosis drug-induced liver injury

**DOI:** 10.3389/fphar.2025.1512815

**Published:** 2025-03-10

**Authors:** Shengfei Pei, Li Yang, Huixia Gao, Yuzhen Liu, Jianhua Lu, Er hei Dai, Chunyan Meng, Fumin Feng, Yuling Wang

**Affiliations:** ^1^ Hebei Coordinated Innovation Center of Occupational Health and Safety, School of Public Health, North China University of Science of Technology, Tangshan, China; ^2^ Hebei Key Laboratory of Immune Mechanism of Major Infectious Diseases and New Technology of Diagnosis and Treatment, The Fifth Hospital of Shijiazhuang, Shijiazhuang, China

**Keywords:** pulmonary tuberculosis, anti-TB therapy, microbiome dysbiosis, probiotics, drug-induced liver injury

## Abstract

**Background:**

This study aimed to explore the distinct characteristics of the gut microbiota in tuberculosis (TB) patients who experienced liver injury following anti-TB treatment compared with those who did not.

**Method:**

We employed a nested case-control study design, recruiting newly diagnosed pulmonary TB patients at Tangshan Infectious Disease Hospital. Participants were categorized into the Antituberculosis Drug-Induced Liver Injury (ADLI) group and the Non-ADLI group based on the occurrence of liver injury after treatment. Both groups received identical anti-TB regimens. Stool samples were collected from patients who developed liver injury within 2–3 weeks of starting treatment, alongside matched controls during the same timeframe. The samples underwent 16S rDNA sequencing, and clinical data and blood samples were also collected for further analysis. At the same time, we constructed mouse models to explore the effects of different anti-tuberculosis drugs on gut microbiota.

**Results:**

Following anti-TB treatment, we observed a decrease in microbial diversity and significant structural changes in the gut microbiota of TB patients (P < 0.05). At T1, the Non_ADLI_T1 group presented relatively high levels of *Phascolarctobacterium*, *Anaerofustis* and *Mailhella*. In contrast, the ADLI_ T1 group presented elevated levels of *Bacteroides*, *Veillonella*, *Clavibacter*, *Corynebacterium*, *Anaerococcus*, *Gardnerella*, *Peptostreptococcus* and *Lautropia*. At T2, the ADLI_T2 group presented increased levels of *Enterococcus*, *Faecalibacterium*, *unclassified_f__Burkholderiaceae*, *Cardiobacterium*, *Ruminococcus_gnavus_group* and *Tyzzerella_4* than did the Non_ADLI_T2 group. Additionally, the ADLI_T2 group presented decreased levels of *Prevotella_9*, *Akkermansia*, *Erysipelotrichaceae_UCG-003, Rubrobacter* and *norank_f__Desulfovibrionaceae* than did the Non_ADLI_T2 group. In animal experiments, similar changes to those in the human population were observed in the mouse model compared to the control group. Any single anti-tuberculosis drug or two-drug combination or three-drug combination can cause dysbiosis of the mouse gut microbiota. The signature genera between groups are different and related to the type of anti-tuberculosis drug.

**Conclusion:**

Anti-tuberculosis treatment induces dysbiosis in the gut microbiota of TB patients. Notably, there are significant differences in microbiota characteristics between TB patients with and without liver injury at both onset and during treatment. There are some differences in the characteristics of bacterial flora in liver injury caused by different drugs.

## 1 Introduction

Tuberculosis (TB) remains a major global health concern, posing a significant threat to public health (Brown et al., 2015). A primary obstacle to effective TB treatment is the occurrence of adverse drug reactions, particularly anti-TB drug-induced liver injury (ADLI), which can have severe consequences (Lim et al., 2023). The complex nature of anti-TB therapy, which often involves multiple drugs administered simultaneously, further complicates our understanding of the mechanisms underlying ADLI. The current understanding suggests that ADLI pathogenesis involves a combination of factors, including inflammatory responses, oxidative stress, immune reactions, and individual genetic variations (Ezhilarasan, 2023). Although preliminary studies have established a connection between the gut microbiota and drug-induced liver injury, research on the role of the gut microbiota in ADLI is still in its infancy.

Recent advances in gut microbiota research have led some scholars to propose that alterations in the gut microbiota may play a role in the development of ADLI. The successful application of fecal microbiota transplantation, probiotics, or prebiotic therapy in certain liver diseases suggests that targeting the gut microbiome could be a promising therapeutic approach for ADLI. However, treatment options for ADLI are currently limited (Chu et al., 2023). Apart from N-acetylcysteine for acetaminophen-induced liver injury, effective treatment options for the vast majority of drug-induced liver injuries remain elusive. Probiotic therapy has shown initial promise in improving liver injury. For example, *Akkermansia* has been shown to ameliorate acetaminophen-induced liver injury through the regulation of short-chain fatty acids (Xia et al., 2022), whereas *Lactobacillus casei* can mitigate isoniazid- and rifampicin-induced liver injury by activating the TLR4/Nf-kB signaling pathway (Guo et al., 2023). Although preliminary studies have established a connection between the gut microbiota and drug-induced liver injury, research on the role of the gut microbiota in ADLI is still in its infancy (Kim et al., 2024; Hao et al., 2023). Most current investigations have focused on the relationship between ADLI and the gut microbiota in experimental animal models.Currently, there is no research focusing on whether the characteristics of intestinal flora after liver damage caused by different anti-tuberculosis drugs are different. The alterations in gut microbiota during anti-tuberculosis treatment have been the subject of investigation in various studies involving tuberculosis patients. In a notable cross-sectional study, Meng examined the differences in gut microbiota profiles between 41 patients with active pulmonary tuberculosis and 28 patients who had undergone 2 months of treatment with HRZE (Meng et al., 2022). Additionally, three other clinical studies have reported that standard anti-tuberculosis chemotherapy can induce significant dysbiosis in gut microbiota (Han et al., 2024; Hu et al., 2019; Wipperman et al., 2017; Maji et al., 2018). However, these investigations did not specifically address the gut microbiota characteristics in patients experiencing liver injury during anti-tuberculosis treatment. Furthermore, the relatively small sample sizes in these studies may limit their ability to capture the full spectrum of microbiota changes associated with anti-tuberculosis therapy. Therefore, it is essential to further explore the gut microbiota changes of ADLI.

This study aims to address two primary objectives: first, to compare the gut microbiota characteristics between pulmonary tuberculosis patients with liver injury and those without during anti-tuberculosis treatment; and second, to analyze the differences in microbiota profiles following liver injury induced by various anti-tuberculosis medications in a mouse model of liver injury.

## 2 Research methods

### 2.1 Survey subjects

This study was designed as a cohort investigation focusing on a specific population of pulmonary TB patients at Tangshan Infectious Disease Hospital, commencing in 2018, with a follow-up period of 3 months. The primary objective was to assess adverse drug reactions and patient prognosis during anti-TB treatment. Stool samples were collected from newly diagnosed patients with pulmonary TB between January 2019 and December 2020. The study encompassed two key time points: T1, which represents specimens collected from patients prior to the initiation of anti-TB treatment upon admission, and T2, which refers to specimens from patients who developed liver injury following anti-TB treatment. Concurrently, matched pulmonary TB patients without liver injury were selected as control subjects. In this study, a total of 400 fecal samples were collected, including 100 samples from the ADLI group and 100 samples from Non-ADLI controls, both before (T1) and after (T2) anti-TB therapy.

#### 2.1.1 Inclusion criteria

(a) Patients must be newly diagnosed with tuberculosis according to the WS 288–2017 Diagnosis of Pulmonary Tuberculosis standard. (b) Patients must be over 18 years of age. (c) Patients must have been administered a daily standard 2HRZE/4HR regimen (H = isoniazid, R = rifampicin, Z = pyrazinamide, E = ethambutol) during the study period. (d) Patients had not used antibiotics, gastrointestinal motility drugs, or microecological regulators in the past month. (e) All patients must have provided informed consent, demonstrating their willingness to participate independently and their ability to adhere to close follow-ups.

#### 2.1.2 The diagnosis of TB was established on the basis of

(a) Moderate or strong positivity in purified protein derivative tests coupled with imaging features indicative of TB. (b) Positive results from interferon-γ release assays alongside imaging features consistent with TB. (c) Positive anti-TB antibody (IgM) tests in conjunction with TB imaging features. (d) Lung histopathology revealing TB lesions, supported by imaging findings.

#### 2.1.3 Exclusion criteria

(a) Patients with comorbidities that could confound results, such as severe heart, brain, lung, liver, and renal diseases; chronic inflammatory conditions; autoimmune diseases; diabetes; and gastrointestinal disorders. (b) Patients whose clinical status changed during anti-TB treatment or who utilized antibiotics other than TB medications.

This study received ethical approval from the School of Public Health at North China University of Science and Technology (approval no. 2017553), and informed consent was obtained from all participants and their families.

#### 2.1.4 Adverse drug-induced liver injury (ADLI) criteria

On the basis of liver enzyme results as defined by the American Thoracic Society, ADLI was classified under the following criteria: (a) an increase of more than threefold in serum alanine transaminase (ALT) or aspartate aminotransferase (AST) compared with the upper limit of normal (ULN), accompanied by symptoms of liver injury. (b) An increase of more than twofold in the serum total bilirubin (TBIL) level compared with that of the ULN, also in the presence of symptoms. (c) The increase in the serum ALT, AST, and TBIL levels was fivefold greater than that in the ULN, regardless of the presence of symptoms.

### 2.2 Data collection

Essential demographic and clinical information, including age, sex, body mass index (BMI), and alcohol consumption history, was gathered from the research subjects. Additionally, the clinical manifestations and status of liver injury were comprehensively documented.

### 2.3 Specimen collection

Stool samples were collected from patients in the morning after they defecated into sterile fecal containers. Using sterile toothpicks, approximately 0.2 g of feces was extracted from the middle portion of each sample and transferred into sterilized cryogenic tubes. Three aliquots were prepared for each sample and immediately stored at −80°C after proper packaging.

Additionally, a fasting venous blood sample of 5 mL was drawn from the antecubital vein between 8:00 a.m. and 9:00 a.m. The blood was allowed to stand at room temperature for 30 min before centrifugation at 3500 RPM for 10 min. The separated serum was then packaged and stored at −80°C for further analysis.

### 2.4 Animal experimental design

Male C57BL/6 mice aged 6–8 weeks (production license: SYXK-(2020-0005)) were purchased from Henan Skbase Biotechnology Co., Ltd, and maintained at the SPF Barrier of the Laboratory Animal Department of North China University of Science and Technology (Ethics number: 2023--SY-052).

A total of 64 mice were randomly assigned to eight groups (n = 8 per group) using a computer-based random order generator. The groups were: control (CON), isoniazid (H), rifampicin (R), pyrazinamide (Z), isoniazid + pyrazinamide (HZ), rifampicin + pyrazinamide (RZ), isoniazid + rifampicin (HR), and isoniazid + rifampicin + pyrazinamide (HRZ). All mice were administered treatments via oral gavage for 14 days as follows: (1) CON group: normal saline; (2) H group: isoniazid (75 mg/kg/day); (3) R group: rifampicin (150 mg/kg/day); (4) Z group: pyrazinamide (375 mg/kg/day); (5) HZ group: isoniazid (75 mg/kg/day) and pyrazinamide (375 mg/kg/day); (6) RZ group: rifampicin (150 mg/kg/day) and pyrazinamide (375 mg/kg/day); (7) HR group: isoniazid (75 mg/kg/day) and rifampicin (150 mg/kg/day); and (8) HRZ group: isoniazid (75 mg/kg/day), rifampicin (150 mg/kg/day), and pyrazinamide (375 mg/kg/day). Four samples from each group were selected for subsequent 16S rDNA gene detection.

### 2.5 Histopathology

Fresh liver tissues were collected and immediately immersed in 4% paraformaldehyde for fixation. Following fixation, the tissues were dehydrated with alcohol, after which they were embedded in paraffin. The embedded tissues were then sliced into thin sections and stained with hematoxylin and eosin for histological examination.

### 2.6 16S rDNA

Faecal samples collected from the mice were rapidly frozen in liquid nitrogen and stored at −80°C. Fecal samples were sent to Meiji Biological Technology Co., Ltd., for analysis. A Fast DNA SPIN kit (MP Biomedicals, California, United States) was used to extract stool DNA. Polymerase chain reaction (PCR) amplification of the V3-V4 region of the bacterial 16S rRNA gene was performed using barcode-indexed primers (338F and 806R) and TransStartFastPfu DNA Polymerase (TransGen, Beijing, China) in an ABI GeneAmp instrument (ABI, United States). The amplicons were then purified by gel extraction (AxyPrep DNA Gel Extraction Kit, Axygen, California, United States) and quantified using a QuantiFluor-ST instrument (Promega, United States). The purified amplicons were pooled in equimolar concentrations, and paired-end sequencing was performed using an Illumina MiSeq system (Illumina, California, United States).

### 2.7 Enzyme-linked immunosorbent assay (ELISA)

Serum levels of TNF-α, IL-6 and IL-1β were analyzed using ELISA kits (Nanjing Jiancheng Bioengineering Institute) according to the instructions of the manufacturer.

### 2.8 Statistical analysis

Data processing and analysis were performed via the R language package and SPSS 22.0 software. Independent sample t-tests and one-way analysis of variance (ANOVA) were used for normally distributed data, whereas nonparametric tests were employed for nonnormally distributed data. The enumeration data are expressed as rates or composition ratios, with differences assessed via the *χ* ^2^ test. Spearman’s rank correlation test was used to assess relationships between variables. Alpha diversity was analyzed via the Wilcoxon rank-sum test, whereas adonis analysis was employed for beta diversity to examine overall microbial community structure differences between groups. To identify differentially enriched taxa in intestinal microbial communities between groups, we utilized linear discriminant analysis effect size (LEfSe). Taxa with linear discriminant analysis (LDA) scores greater than three and P values less than 0.01 or 0.05 were considered statistically significant. Furthermore, we assessed correlations between the intestinal microbiota and clinical biochemical indices via Spearman’s correlation coefficient analysis.

## 3 Results

### 3.1 Basic characteristics of the study subjects

In this study, a total of 400 fecal samples were collected, including 100 samples from the ADLI group and 100 samples from Non-ADLI controls, both before (T1) and after (T2) anti-TB therapy. As depicted in Table 1, there were no statistically significant differences in age, sex, BMI, marital status, residence, smoking status, or alcohol consumption between the liverinjury and non liver injury groups (all P > 0.05).

**TABLE 1:** Baseline clinical characteristics of the study subjects.

Indicator		Non-ADLI (100)		ADLI(100)		χ^2^	P
		N	%	N	%		
Age	18–44	50	50.00	42	42.00	2.266	0.322
	45–60	29	29.00	39	39.00		
	≥61	21	21.00	19	19.00		
Sex	Man	70	70.00	72	72.00	0.097	0.755
	Woman	30	30.00	28	28.00		
BMI(kg/m^2^)	≤18.4	9	9.00	8	8.00	1.536	0.464
	18.5–23.9	63	63.00	71	71.00		
	24–27	28	28.00	21	21.00		
Marriage	No	32	32.00	26	26.00	0.874	0.350
	Yes	68	68.00	74	74.00		
Address	Cities	45	45.00	36	36.00	1.681	0.195
	Village	55	55.00	64	64.00		
Somking	No	63	63.00	58	58.00	0.523	0.470
	Yes	37	37.00	42	42.00		
Drinking	No	70	62.50	62	68.75	1.426	0.232
	Yes	30	37.50	38	31.25		

### 3.2 Changes in serum proinflammatory cytokine levels

Changes in the serum levels of the proinflammatory cytokines TNF-α, IL-6, and IL-1β in the two groups of pulmonary tuberculosis patients before and after antituberculosis treatment were detected via ELISA. As depicted in Table 2, when comparing the groups, only TNF-α was significantly different at T1. However, at T2, the expression levels of inflammatory cytokines in the ADLI group were greater than those in the non-ADLI group. In the longitudinal comparison, there was no statistically significant difference between T1 and T2 in the non-ADLI group (P > 0.05), whereas in the ADLI group, the expression levels of inflammatory cytokines at T2 were greater than those at T1 (P < 0.05).

**TABLE 2:** Changes in serum inflammatory factor levels in the non-ADLI group and ADLI group.

	Non-ADLI-T1	Non-ADLI-T2	ADLI-T1	ADLI-T2
TNF-α	133.04 ± 33.64	164.02 ± 45.56	195.05 ± 54.14^(c)^	304.67 ± 113.47^(ab)^
IL-6	4.44 ± 1.60	4.57 ± 1.25	5.792 ± 2.37	15.41 ± 6.40^(ab)^
IL-1β	11.79 ± 1.91	12.06 ± 2.02	10.91 ± 1.46	21.29 ± 5.90^(ab)^

Note:

^a^ Compared with Non-ADLI-T2.

^b^ Compared with ADLI-T1.

^c^ Compared with Non-ADLI-T1, all P < 0.05.

### 3.3 Analysis of intestinal flora characteristics in patients with pulmonary tuberculosis

#### 3.3.1 Comparative analysis of diversity

**FIGURE 1:**
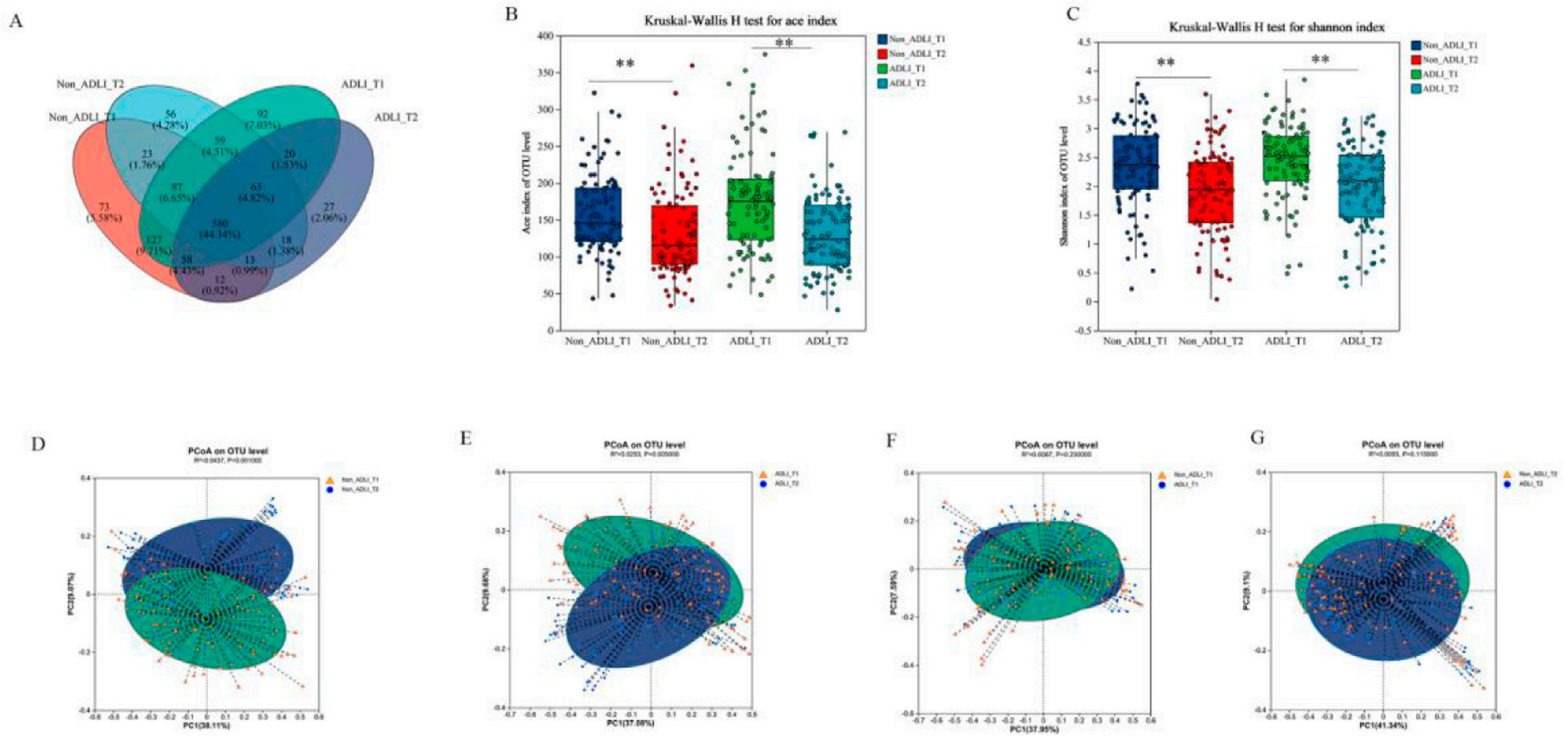
Changes in the microbiota diversity of tuberculosis patients. Notes: Non_ADLI_T1—nonsevere-damaged group at T1; Non_ADLI_T2—nonseiver-damaged group at T2; ADLI_T1—liver-damaged group at T1; ADLI_T2—liver-damaged group at T2; *: (P < 0.05), **: (P < 0.01), ***: (P < 0.001). **(A)**: Composition of each group at the OTU level. **(B, C)**: α diversity in the Non-ADLI and ADLI groups from T1 to T2. D-G Weighted pCoA analysis of the Non-ADLI and ADLI groups at T1 to T2. **(D)**: Non_ADLI_T1 vs T2, **(E)**: ADLIT1 vs T2, **(F)**: Non_ADLIT1 vs ADLI_T1, **(G)**: Non-ADLI_T2 vs. ADLIT2 (P < 0.05).

We used the Kruskal-Wallis test to analyze changes in the alpha diversity of the gut microbiota between the ADLI and non-ADLI groups. (Figures 1A–C). Initially, when comparing the T1 and T2 time points, the ACE indices, which are used to measure community richness, significantly decreased in both the Non-ADLI and ADLI groups (P < 0.05) (Figure 1B). Additionally, the Simpson indices were significantly different between the ADLI and non-ADLI groups from T1 to T2 (P < 0.05). These results indicate that from T1 to T2, both the Non-ADLI and ADLI groups presented a decreasing trend in the richness and diversity of their gut microbiota following antituberculosis treatment (Figure 1C). In the intergroup comparisons at the same time points, as illustrated in Figures 1B, C, no statistically significant differences were detected in the ACE indices used to assess community richness. Similarly, there were no statistically significant differences in the Simpson indices reflecting community diversity (P > 0.05). These results suggest that the influence of antituberculosis treatment on the abundance and diversity of the gut microbiota is comparable in both the non-ADLI and ADLI groups.

Principal coordinate analysis (PCoA), based on the weighted UniFrac distance matrix, was used to visualize the structural differences in microbiota among the groups, and the Adonis test was employed to statistically assess these differences. First, in the longitudinal comparative analysis, both the ADLI and non-ADLI groups exhibited statistically significant differences in the analysis based on weighted UniFrac distance matrices from the T1 to T2 time points (both P < 0.05,ADLI T1vsT2: R^2^ = 0.0437, non-ADLI T1vsT2: R^2^ = 0.0253). These results indicate that the microbiota structures of both the ADLI and non-ADLI groups changed from T1 to T2 after receiving antituberculosis treatment (Figures 1D, E). There were no statistically significant differences between the ADLI and non-ADLI groups at the T1 and T2 time points (T1: P = 0.23, T2: P = 0.115) (Figures 1F, G).

#### 3.3.2 Phylum and genus composition of each group

**FIGURE 2:**
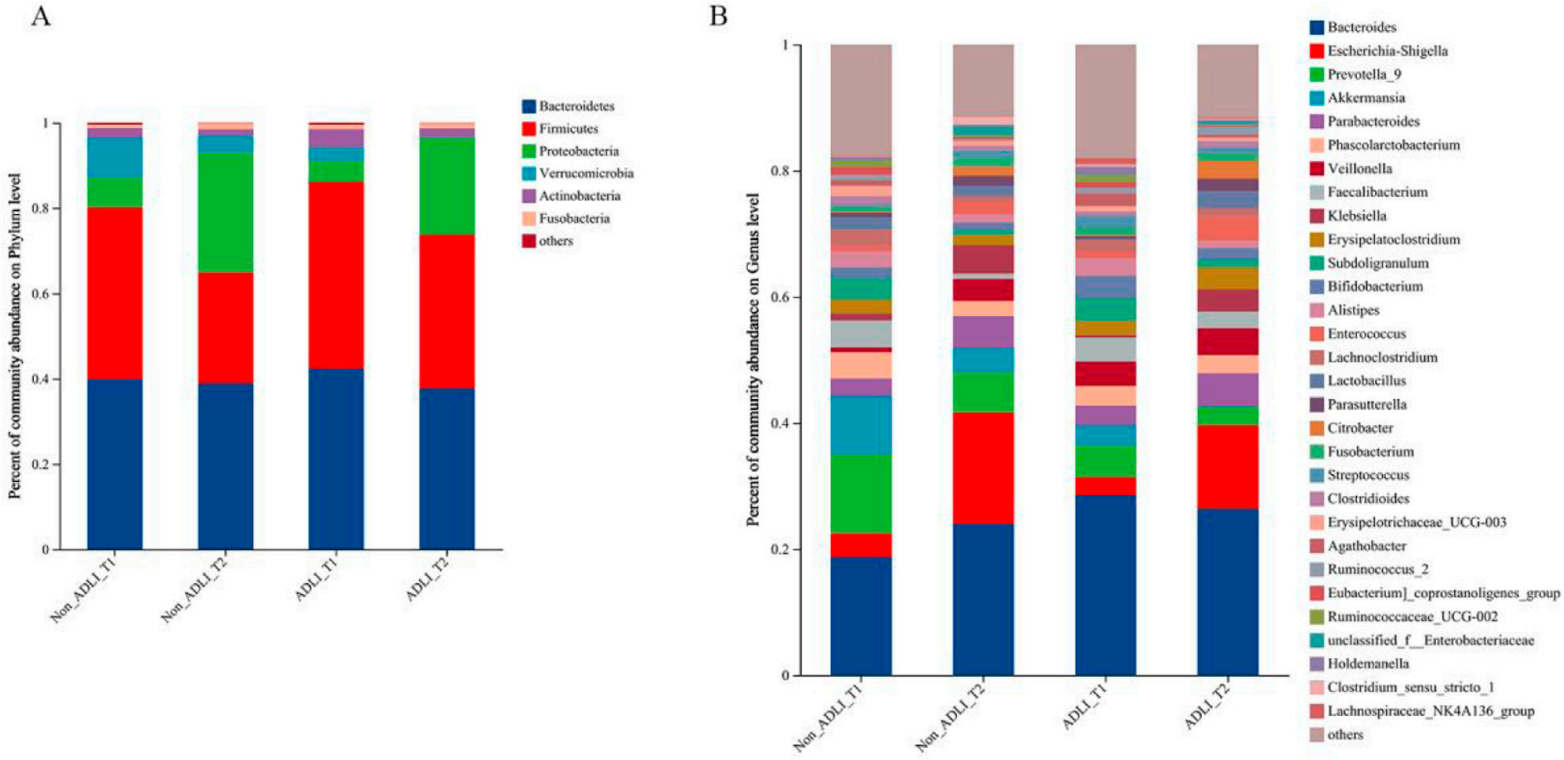
Composition of phylum and genus in patients with pulmonary tuberculosis. Notes: Non_ADLI_T1—nonsevere-damaged group at T1; Non_ADLI_T2—nonseiver-damaged group at T2; ADLI_T1—liver-damaged group at T1; ADLI_T2—liver-damaged group at T2. **(A)**: The composition of the four groups at the phylum level. **(B)**: The composition of the four groups at the genus level.

A total of 400 fecal samples were collected for sequencing. At the phylum level (Figure 2A), the majority of the OTUs belonged to the phyla *Bacteroidetes* and *Firmicutes*, followed by *Proteobacteria*, *Verrucomicrobia*, *Actinobacteria* and *Fusobacteria*. At the genus level (Figure 2B), the gut microbiota in the four groups was mainly composed of 30 genera. Specifically, the top 10 genera included *Bacteroides*, *Escherichia-Shigella*, *Prevotella_9*, *Akkermansia*, *Parabacteroides*, *Phascolarctobacterium*, *Veillonella*, *Faecalibacterium*, *Klebsiella*, and *Erysipelatoclostridium*. Among the four groups, *Bacteroides* accounted for approximately 20% of the total sequences in each group and was the dominant genus.

#### 3.3.3 Lefse analysis of the different bacterial genera in each group

Further analysis of the differences in microbial communities between groups was conducted via LEfSe analysis to examine the differential microbial taxa between different groups, and linear discriminant analysis (LDA) was employed to assess the magnitude of the effects of different species.

**FIGURE 3:**
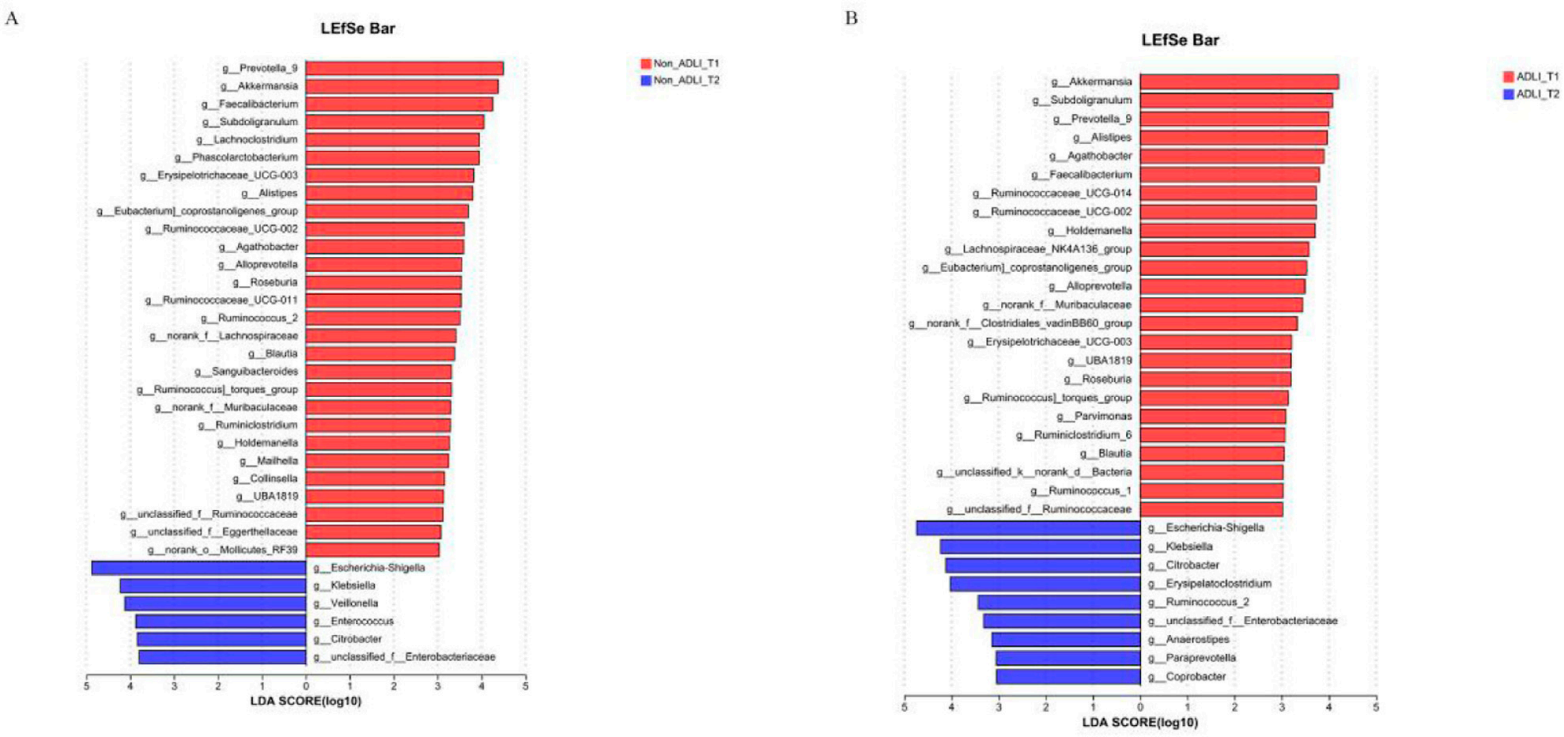
Longitudinal comparison of bacterial genera with and without liver injury in patients with pulmonary tuberculosis. Notes: Non_ADLI_T1—nonsevere-damaged group at T1; Non_ADLI_T2—nonseiver-damaged group at T2; ADLI_T1—liver-damaged group at T1; ADLI_T2—liver-damaged group at T2. **(A)** Characteristics of genus changes from T1 to T2 in Non_ADLI group, **(B)** Characteristics of genus changes from T1 to T2 in ADLI group.

The longitudinal comparison analysis initially revealed that there were a total of 34 differentially abundant taxa between the T1 and T2 time points in the Non-ADLI group. (Figure 3A). Compared with the Non-ADLI_T1 group, the Non-ADLI_T2 group presented a greater abundance of a total of 28 genera, with *Prevotella_9*, *Akkermansia*, *Faecalibacterium*, *Subdoligranulum* and *Lachnoclostridium* being the top 5. The relative abundances of *Escherichia-Shigella*, *Klebsiella*, *Veillonella*, *Enterococcus*, *Citrobacter* and *unclassified_f__Enterobacteriaceae* were lower in the Non_ADLI_T2 group (Figure 3A).

In the ADLI group, 33 differentially abundant taxa were identified between the T1 and T2 time points. Compared with those in the ADLI_T1 group, a total of 24 genera were more abundant in the ADLI_T2 group, with the top five being *Akkermansia*, *Subdoligranulum*, *Prevotella_9*, *Alistipes* and *Agathobacter* (Figure 3B). The relative abundances of *Escherichia-Shigella*, *Klebsiella*, *Citrobacter*, *Erysipelatoclostridium*, *Ruminococcus_2*, *unclassified_f__Enterobacteriaceae*, *Anaerostipes*, *Paraprevotella* and *Coprobacter* were lower (Figure 3B).

**FIGURE 4:**
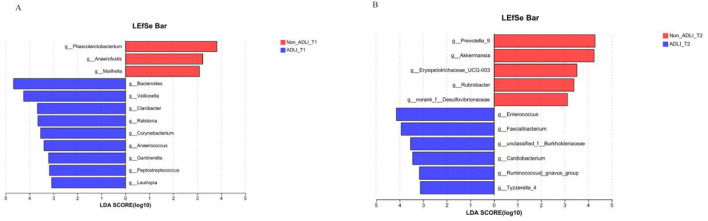
Comparative analysis of bacterial genera at the same time point between pulmonary tuberculosis patients with liver injury and those without liver injury. Notes: Non_ADLI_T1—nonsevere-damaged group at T1; Non_ADLI_T2—nonseiver-damaged group at T2; ADLI_T1—liver-damaged group at T1; ADLI_T2—liver-damaged group at T2. **(A)**: Characteristics of genus differences between Non_ADLI group and ADLI group at T1. **(B)**: Characteristics of genus differences between Non_ADLI group and ADLI group at T2.

The comparison at the same time point indicates that initially, at the T1 time point, a comparison was conducted between Non-ADLI and ADLI using LDA >3. The results revealed differences in 12 genera between the two groups. The relative abundances of *Phascolarctobacterium*, *Anaerofustis* and *Mailhella* were greater in the Non_ADLI_T1 group, whereas *Bacteroides*, *Veillonella*, *Clavibacter*, *Corynebacterium*, *Anaerococcus*, *Gardnerella*, *Peptostreptococcus* and *Lautropia* were more abundant in the ADLI_T1 group (Figure 4A). At the T2 time point, 11 genera differed between the Non-ADLI_T2 and ADLI_T2 groups. The relative abundances of *Enterococcus*, *Faecalibacterium*, *unclassified_f__Burkholderiaceae*, *Cardiobacterium*, *Ruminococcus gnavus* and Tyzzerella_4 were greater in the ADLI_T2 group than in the Non_ADLI_T2 group. Conversely, *Prevotella_9*, *Akkermansia*, *Erysipelotrichaceae_UCG-003*, *Rubrobacter* and *norank_f__Desulfovibrionaceae* were less abundant in the ADLI_T2 group than in the Non_ADLI_T2 group (Figure 4B).

**FIGURE 5:**
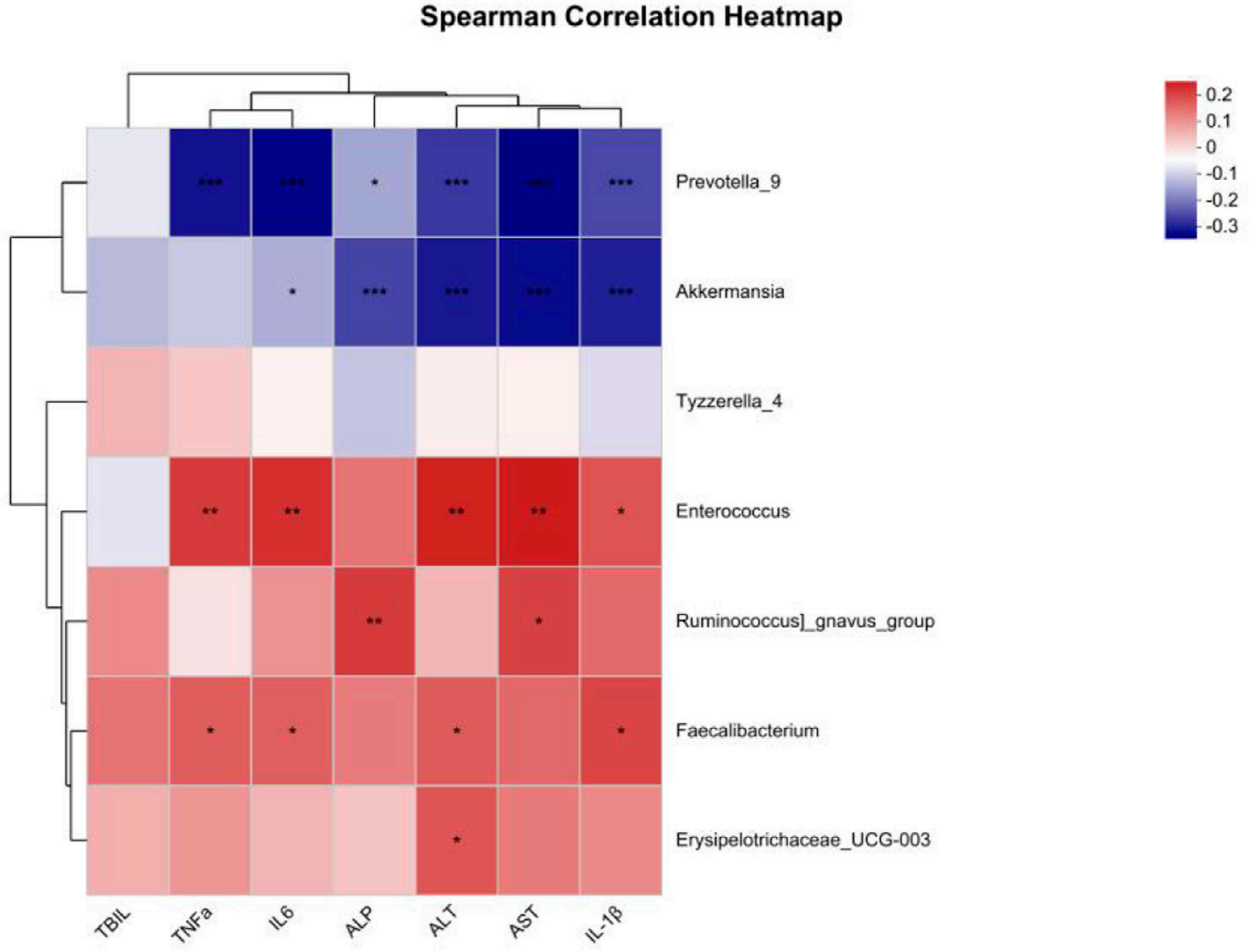
Correlation analysis between different bacterial genera and clinical indicators. The color shading represents the degree of correlation, with blue representing a negative correlation and red representing a positive correlation; *: P < 0.05; **: P < 0.01; ***: P < 0.001.

We conducted a correlation analysis between the differentially abundant taxa at the T2 time point and liver function as well as inflammatory factors. The results are presented in Figure 5. *Prevotella_9* and *Akkermansia* were negatively correlated with the liver function markers ALT and AST (P < 0.05), whereas *Enterococcus*, *Faecalibacterium* and *Erysipelotrichaceae_UCG-003* were positively correlated with ALT. *Enterococcus* and *Ruminococcus_gnavus_group* were positively correlated with AST (P < 0.05). Additionally, *Prevotella_9* was negatively correlated with the inflammatory factors TNF-α, IL-6, and IL-1β, whereas *Akkermansia* was negatively correlated with IL-6 and IL-1β. *Enterococcus* and *Faecalibacterium* were positively correlated with TNF-α, IL-6 and IL-1β (P < 0.05).

### 3.4 Characteristics of the bacterial flora in anti-tuberculosis drug-induced liver injury in mice

Furthermore, we established three mouse models of liver injury induced by first-line antituberculosis drugs to further investigate the differences in microbial profiles between liver injury induced by different antituberculosis drugs.

#### 3.4.1 Pathological changes of mice

**FIGURE 6:**
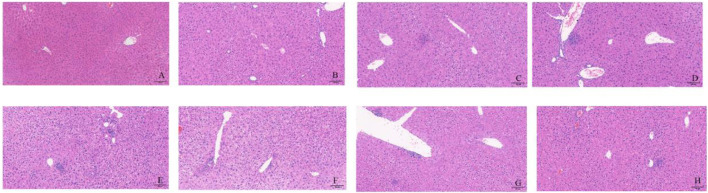
Liver pathological changes in each group of mice. Notes: **(A)**: CON, **(B)**: INH, **(C)**: RIF, **(D)**: PZA, **(E)**: INH+PZA, **(F)**: RIF+PZA, **(G)**: INH+PZA, **(H)**: INH+RIF+PZA. Scaleplate:100 μm.

First, as shown in Figure 6, each anti-tuberculosis drug, HRZ, could lead to the occurrence of liver injury. In the Con group, hepatocytes were arranged radially and orderly around the central lobular vein, with the same cell size, and no inflammatory cell infiltration was observed in the lobular or portal area. In each treatment group, some areas of liver tissue presented loose cytoplasm and ballooning degeneration of hepatocytes, accompanied by punctate necrosis and granular degeneration of hepatocytes, loose and mildly stained cytoplasm, and a small amount of focal infiltration of inflammatory cells in the lobules (Figure 6).

#### 3.4.2 Changes in the characteristics of the intestinal flora in mice

**FIGURE 7:**
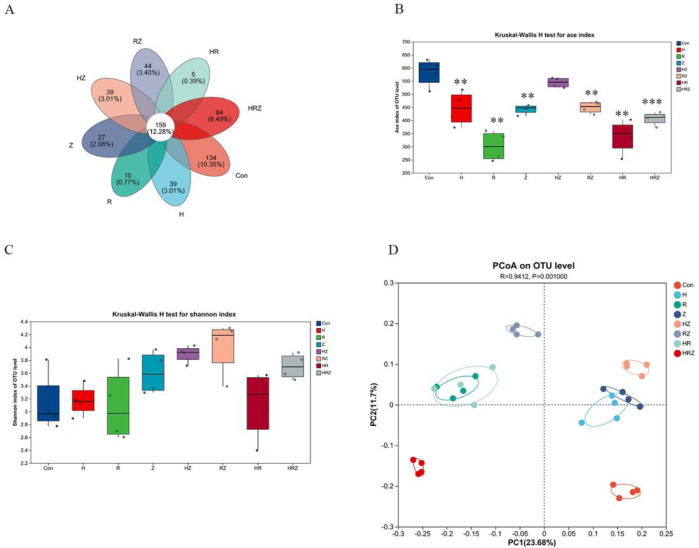
Altered diversity of gut microbiota in mice. Notes: CON: Control, H:INH, R:RIF, Z:PZA, HZ:INH+PZA, RZ:RIF+PZA, HR:INH+RIF, HRZ:INH+RIF+PZA. **(A)** Venn diagram analysis based on OUT level, **(B, C)** represent alterations in alpha diversity: **(B)** ace index, **(C)** shannon index, **(D)** alterations in β diversity in mice.

Figure 7A shows that at the OUT level, the unique OUT species of the bacterial flora after liver injury caused by each drug are different. Unlike in the population, the ACE index, which represents richness in a diversity, decreased in accordance with the population (Figure 7B), but there was no statistically significant difference in the Shannon index (Figure 7C). PCoa analysis revealed that compared with the control group, each group was significantly different from the con group (*R* ^2^ = 0.9412, P = 0.001) (Figure 7D), which suggested that antituberculosis drugs could significantly change the composition of the intestinal flora in mice.

**FIGURE 8:**
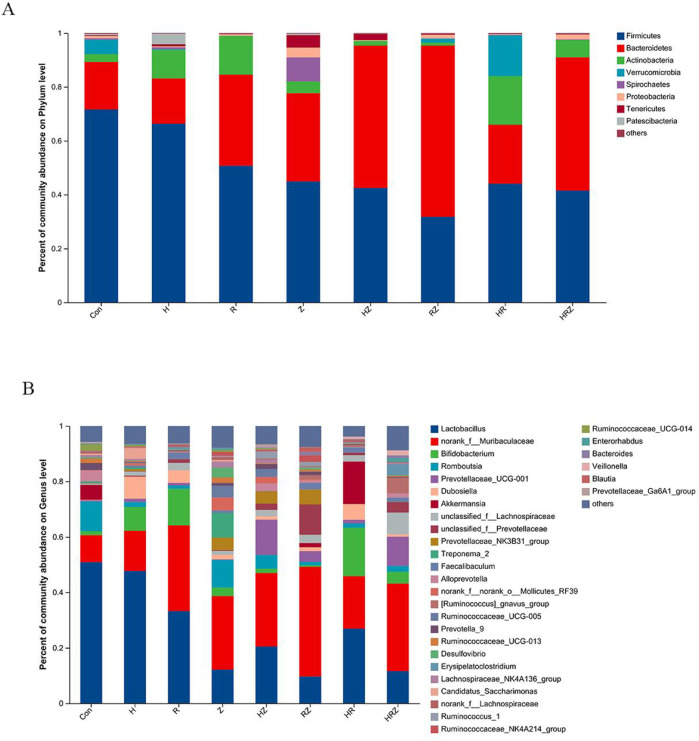
Phyla and genus composition after liver injury induced by different drugs. Notes: CON:Control, H:INH, R:RIF, Z:PZA, HZ:INH+PZA, RZ:RIF+PZA, HR: INH+RIF, HRZ:INH+RIF+PZA. **(A)** Mice at phylum level, **(B)** Mice at genus level.

Some similarity exists between the phyla and genus-level composition of mice and humans, and the top five phyla were *Firmicutes, Bacteroidetes*, *Actinobacteria*, *Verrucomicrobia* and *Spirochaetes* (Figure 8A). The top 10 genera were *Lactobacillus*, *norank_f__Muribaculaceae*, *Bifidobacterium*, *Romboutsia*, *Prevotellaceae_UCG-001*, *Dubosiella*, *Akkermansia*, *unclassified_f__Lachnospiraceae*, *unclassified_f__Prevotellaceae* and u*nclassified_f__Prevotellaceae* (Figure 8B).

**FIGURE 9:**
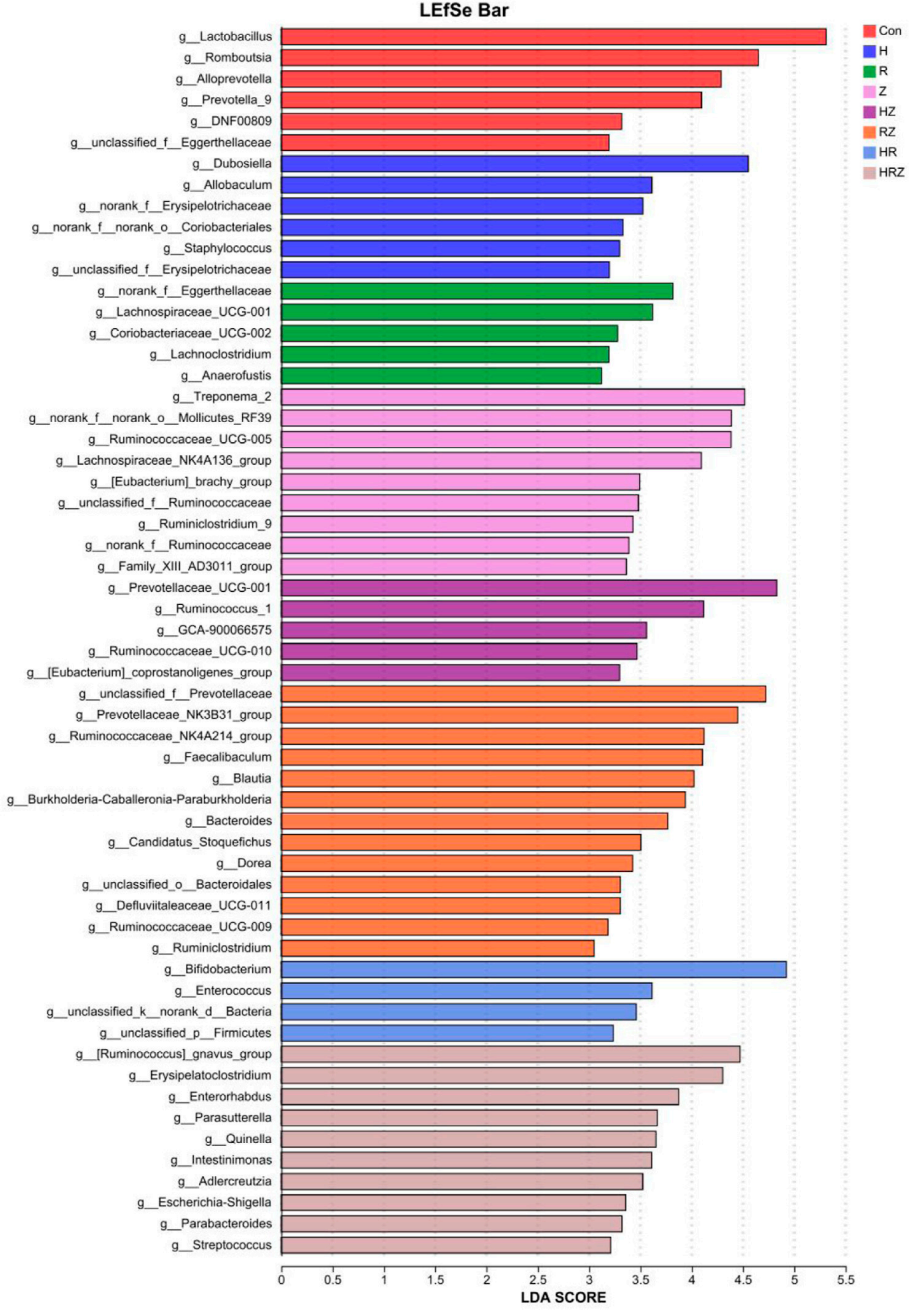
Lefse analysis shows the marker bacterial genera of liver injury induced by different drugs. Notes: CON: Control, H:INH, R:RIF, Z:PZA, HZ:INH+PZA, RZ:RIF+PZA, HR:INH+RIF, HRZ:INH+RIF+PZA.

Figure 9 shows the genera unique to the different groups (LDA > 3, P < 0.05). In the additional file 1, we compared the characteristics of the bacterial genera associated with each drug compared with those associated with the con group in detail and found that the characteristics of bacterial dysbiosis caused by each drug were different, but there were some common characteristics. For example, compared with that in the CON group, *Lactobacillus* was not significantly different in the H group, but *Lactobacillus* was decreased in the other groups. The abundance of *Akkermansia* was greater in the HR group than in the CON group and tended to decrease in each of the other treatment groups. *Bifidobacterium* increased in the H, R and HR treatment groups but did not change in the PZA treatment groups (Z, HZ, RZ, HRZ). All three pairs tested caused significant compositional changes, which again occurred at a different community level than that induced by the whole HRZ. The above experiments indicate that each antibiotic component of HRZ causes overall dysbiosis in mice with anti-TB drug-induced liver injury and that different combinations of components cause different bacterial genus characteristics.

## 4 Discussion

This study investigated alterations in the gut microbiota among pulmonary tuberculosis patients receiving antituberculosis treatment through a nested case‒control design. Concurrently, we established a mouse model to examine the gut microbiota characteristics linked to various types of drug-induced liver injury. This research represents the largest-scale analysis to date comparing gut microbiota changes before and after antituberculosis treatment in patients, specifically those with and without liver injury. By integrating animal experiments, we evaluated the impact of three first-line antituberculosis drugs on the gut microbiota following liver injury. Our findings revealed a significant decrease in gut microbiota diversity during antituberculosis treatment, accompanied by notable structural changes. Distinct differences were observed in the gut microbiota profiles of patients with and without liver injury at the same time points. Each antibiotic component (INH, RIF and PZA) was found to induce liver injury and gut dysbiosis, with varying drug combinations leading to distinct microbial profiles.

In normal physiology, the gut microbiota and the host have a mutually dependent relationship, maintaining a dynamic balance. A decrease in microbial diversity may lead to various diseases (Zhou et al., 2020). Consistent with previous research, our study results demonstrate that antituberculosis treatment leads to a reduction in gut microbiota diversity in patients with pulmonary tuberculosis (Hu et al., 2019). Specifically, we observed a decline in the average relative abundance of the phylum Firmicutes during treatment (Cao et al., 2021).

However, the observed changes in gut microbiota diversity and richness among pulmonary tuberculosis patients contrast with those reported in some animal studies, where antituberculosis drugs were shown to affect Ace richness indices primarily without altering Simpson diversity indices (Li et al., 2023; Namasivayam et al., 2017). Conversely, a recent study indicated that INH-induced liver damage significantly impacts both diversity and richness (Liu et al., 2023). In a rat model of HR-induced liver injury, both the Chao and Shannon indices decreased (Li et al., 2022).

Our animal results revealed a decrease in the Ace index and no significant difference in the Simpson index. We speculate that this may be due to the complexity of the patient population; although we established some inclusion and exclusion criteria, certain medication and dietary factors are uncontrollable during treatment, which may be the primary cause of the differences. Additionally, the results may also be influenced by the limited sample size in animal experiments. Furthermore, we compared the α diversity and β diversity of the two groups of patients before and after antituberculosis treatment at the same time points and found no statistically significant differences, indicating that the occurrence of ADLI may be associated with specific taxa.

Furthermore, we compared the microbial characteristics of the two groups before and after antituberculosis treatment, as well as at the same time points, and found differences in specific taxa between the two groups. In this study, to eliminate potential confounding factors, we matched the participants as closely as possible in terms of age and sex. There were no statistically significant differences between the groups in terms of marital status, BMI, geographical distribution, alcohol consumption, or smoking status, which helped control for potential confounding effects on the gut microbiota composition. When the samples before antituberculosis treatment at T1 were compared, we detected differences in 12 taxa. At T1, the relative abundance of the *Bacteroides* genus was greater in the ADLI group than in the Non-ADLI group. Several studies have shown that the abundance of *Bacteroides* increases after liver injury (Xu et al., 2024). *Bacteroides* species are typically commensal organisms in the gut, but they can become opportunistic pathogens when they reside elsewhere. When the intestinal barrier is compromised (intestinal permeability), *Bacteroides* can translocate across the intestinal mucosa into normally sterile tissues, ultimately leading to different disease states. Additionally, the abundance of *Veillonella* was greater in the ADLI group than in the Non-ADLI group at T1. An increased abundance of *Veillonella* has been associated with certain liver diseases. Patients with PSC show changes in the abundance of certain gut microbiota, particularly an increase in *Veillonella* abundance (Cortez et al., 2020). Whether these differing taxa at T1 are related to the occurrence of injury or noninjury in some pulmonary tuberculosis patients after antituberculosis treatment warrants further investigation.

Additionally, the interactions between the gut microbiome and the immune signaling system may play a crucial role in modulating the inflammatory response. These interactions can either promote anti-inflammatory pathways by stimulating regulatory cells or contribute to pro-inflammatory responses (Somboro et al., 2021). Furthermore, gut microbiota can influence drug metabolism by colonizing the gut and competing for resources, while also providing protection against pathogenic microorganisms (Parada Venegas et al., 2019). Cytokines such as TNF-α, IL-6, and IL-1β have been shown to impact intestinal tight junctions, subsequently affecting the composition of the gut microbiota (Kaminsky et al., 2021; Suzuki et al., 2011). Therefore, we conducted a comparative analysis of the microbial profiles of pulmonary tuberculosis patients with and without liver injury, aiming to explore the association between these microbiota characteristics and inflammatory factors.

At T2, we observed an enrichment of the anti-inflammatory genera *Akkermansia* and *Prevotella_9* in the Non-ADLI group, whereas the proinflammatory genus *Enterococcus* was enriched in the ADLI group. This imbalance of anti-inflammatory and proinflammatory bacteria may partially explain the occurrence of ADLI in some patients with pulmonary tuberculosis during anti-TB treatment. Additionally, at T2, we found that the relative abundance of *Tyzzerella_*4 was greater in the ADLI group than in the Non-ADLI group. Although the literature on *Tyzzerella_4* is limited, several studies have reported its association with diseases. It increases in high-risk cardiovascular disease patients and is associated with increased lifelong risk of cardiovascular disease. Furthermore, *Tyzzerella_4* is increased in liver cancer patients compared with healthy individuals. However, correlation analysis revealed no statistically significant associations between *Tyzzerella_4* and clinical indicators, possibly because of its relatively low abundance in the gut microbiota. The pathogenesis of drug-induced liver injury is characterized by significant inflammatory responses, and the imbalance between anti-inflammatory and pro-inflammatory genera in patients with pulmonary tuberculosis may intensify these inflammatory reactions. Further,we analyzed the different genera present in pulmonary TB patients with and without injury at T2 and their correlations with the inflammatory factors TNF-α, IL-6, and IL-1β. We found that *Prevotella_9* and *Akkermansia* were negatively correlated with TNF-α, IL-6 and IL-1β, whereas *Enterococcus* was positively correlated. Overall, our findings suggest that the occurrence of ADLI may be associated with specific anti-inflammatory genera, such as *Prevotella_9* and *Akkermansia*. Further investigations into their mechanistic roles in ADLI are warranted.

Considering that all pulmonary TB patients received anti-tuberculosis drug treatment, we hypothesized that certain genera might exhibit similarities. Interestingly, in the longitudinal comparison, we found that the genera exhibiting differential changes in both the Non-ADLI and ADLI groups were similar, characterized by a decrease in beneficial bacteria and an increase in opportunistic pathogens. *Akkermansia* and *Prevotella_9* are closely associated with short-chain fatty acid synthesis (Shi et al., 2022). Numerous studies have suggested that a deficiency or reduction in *Akkermansia* abundance is linked to various diseases, such as obesity, diabetes, hepatic steatosis, inflammation, and the response to cancer immunotherapy (Rodrigues et al., 2022; Effendi et al., 2022; Wang et al., 2020; Rao et al., 2021). These findings suggest that *Akkermansia* and *Prevotella_9* may be key genera involved in regulating ADLI and that the occurrence of ADLI seems to be associated with the depletion of these two genera. *Escherichia-Shigella was* significantly enriched in both groups after antituberculosis treatment. Toxins produced by *Escherichia-Shigella* can directly or indirectly damage liver cells (Xin et al., 2022). For example, the endotoxin LPS can activate immune cells in the liver, such as Kupffer cells, leading to the release of proinflammatory cytokines such as TNF-α and IL-6, triggering liver inflammation and injury (Harutyunyan et al., 2020; Lopez-Montoya et al., 2022). Additionally, an animal study suggested that a significant decrease in short-chain fatty acids accompanies isoniazid- and rifampicin-induced liver injury. Our gut analysis results partially support the findings from animal experiments, indicating that the reduction in beneficial bacteria associated with short-chain fatty acids and the increase in opportunistic pathogens may influence the occurrence of ADLI.

For the animal experiments, we compared the effects of HRZ and different combinations of HRZ on the gut microbiota. Each drug can cause dysbiosis of the gut microbiota, and the characteristics of dysbiosis induced by different drugs differ. Interestingly, *Akkermansia* was downregulated during anti-TB treatment in humans, and a similar phenomenon was found in animals. Although the abundance of *Akkermansia* is increased in HR, which may be related to the toxic mechanism of H and R combined, the increased abundance of *Akkermansia* may be related to its antioxidative stress properties. Moreover, the abundance of Bifidobacterium increased in the H, R and HR treatment groups but did not change in the PZA treatment groups (Z, HZ, RZ or HRZ). Whether this effect is related to the properties of PZA-induced liver injury warrants further exploration.

Compared with previous studies, our study was conducted with a larger sample size (Hu et al., 2019; Ye et al., 2022; Wu et al., 2023; Seraphin et al., 2023). We conducted an extensive comparison of changes in the gut microbiota between pulmonary tuberculosis patients who experienced liver injury and those who did not during antituberculosis treatment. However, our study has certain limitations. First, the study population was limited to a single center, which may limit generalizability. Nonetheless, the larger sample size compared to earlier studies enhances the statistical power of our results. Future research should consider multi-center studies to further investigate the alterations in intestinal flora among tuberculosis patients with and without liver injury across different regions and ethnic groups. Second, implementing standardized diets for all participants poses challenges that may impact the composition of the gut microbiota. Dietary content and patterns are recognized as significant factors influencing gut microbiota composition. Future studies should explore whether these changes in gut microbiota can be assessed through standardized dietary interventions. Third, we analyzed the gut microbiota from stool samples rather than from intestinal samples, which may not fully represent the entire gut microbiota landscape. Fourth, while the standard antituberculosis treatment includes HRZE, our research did not incorporate EMB in animal experiments. Given the existing results, it is plausible that EMB significantly impacts the intestinal flora, warranting further studies on this topic.

## 5 Conclusion

We performed a comprehensive analysis of gut microbiota changes in pulmonary tuberculosis patients before and after antituberculosis treatment and performed a detailed comparison of microbiome characteristics between those with and without liver injury. Additionally, we explored the correlations between various bacterial genera and liver function indicators following liver injury. This study highlights the profound influence of antituberculosis treatment on the gut microbiota of TB patients, emphasizing the distinct microbiome profiles observed in patients with and without ADLI, both at the onset of treatment and during its course. Our findings indicate that *Akkermansia* and *Prevotella_9* may play significant roles in adverse drug-induced liver injury (ADLI). Future research should further investigate the relationships between these genera and ADLI from an animal model perspective. These findings could pave the way for novel therapeutic strategies targeting the gut microbiome to mitigate ADLI and improve outcomes for TB patients.

## 6 Lay summary

In patients with pulmonary tuberculosis receiving anti-TB treatment, we discovered that certain gut bacteria are linked to the development of ADLI. Understanding and modifying these factors could help mitigate the adverse effects associated with anti-TB therapy.

## Data Availability

The raw sequencing data generated in this study have been deposited in the NCBI BioProject database under accession number PRJNA1226094.
