# Supplementary material for: The association between the gut microbiome and antituberculosis drug-induced liver injury
Source: Front Pharmacol. 2025 Mar 10;16:1512815. doi: 10.3389/fphar.2025.1512815 (PMC11931021; doi:10.3389/fphar.2025.1512815)
Supplement: Supplementary file 1 [file DataSheet1.docx]

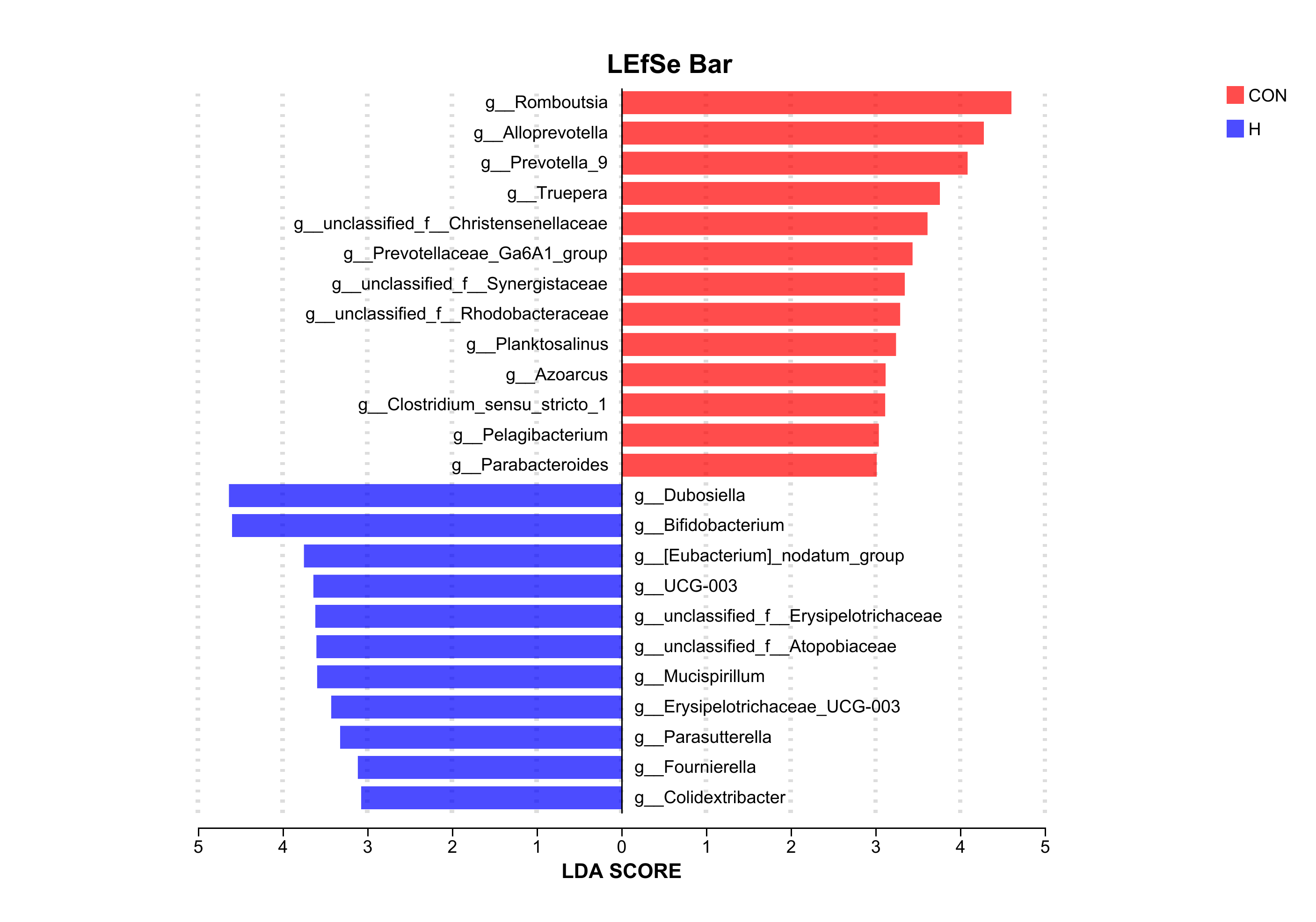


Figure S1: Genus differences in INH alone compared to control


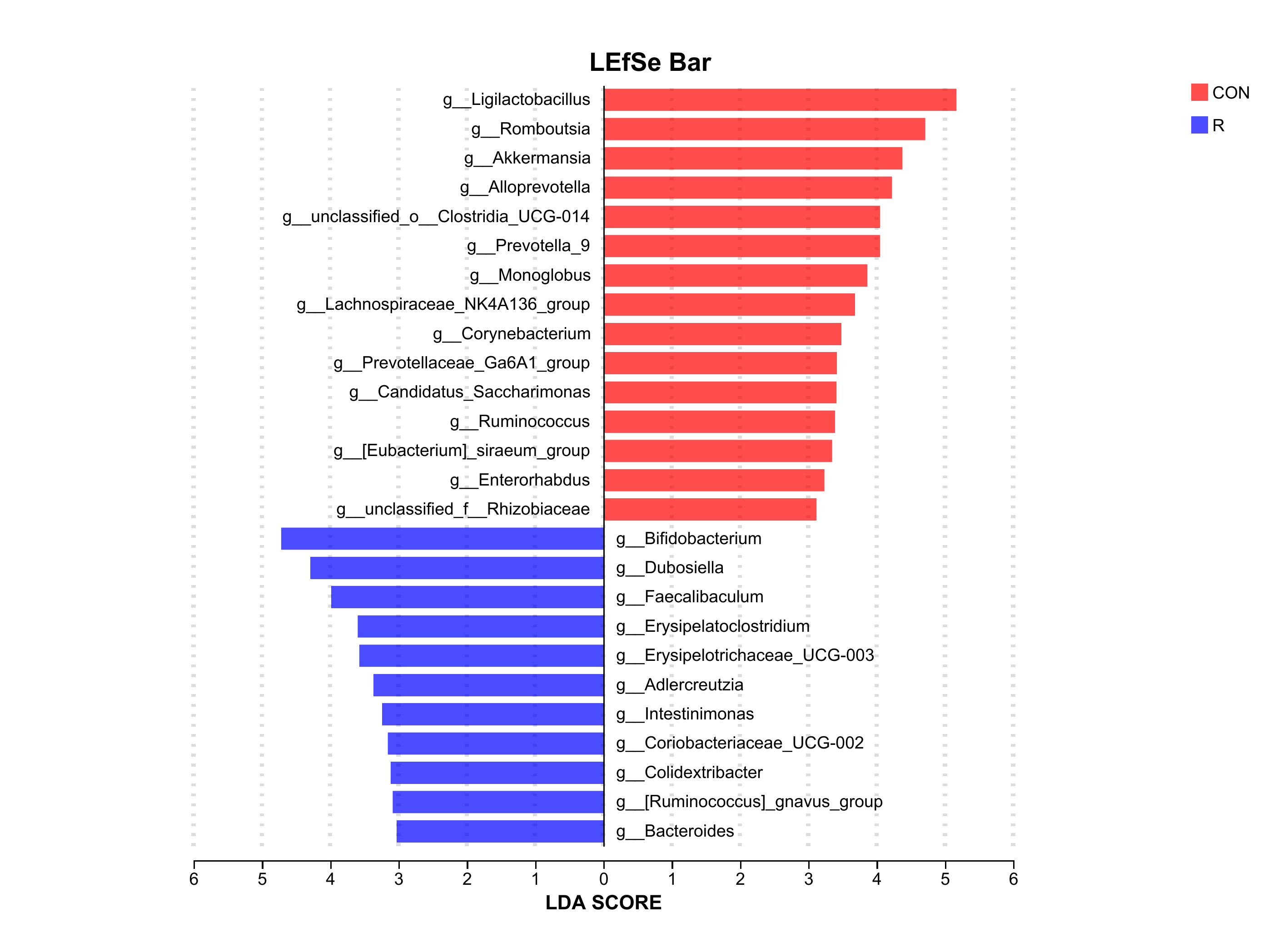


Figure S2: Genus differences in RIF alone compared to control


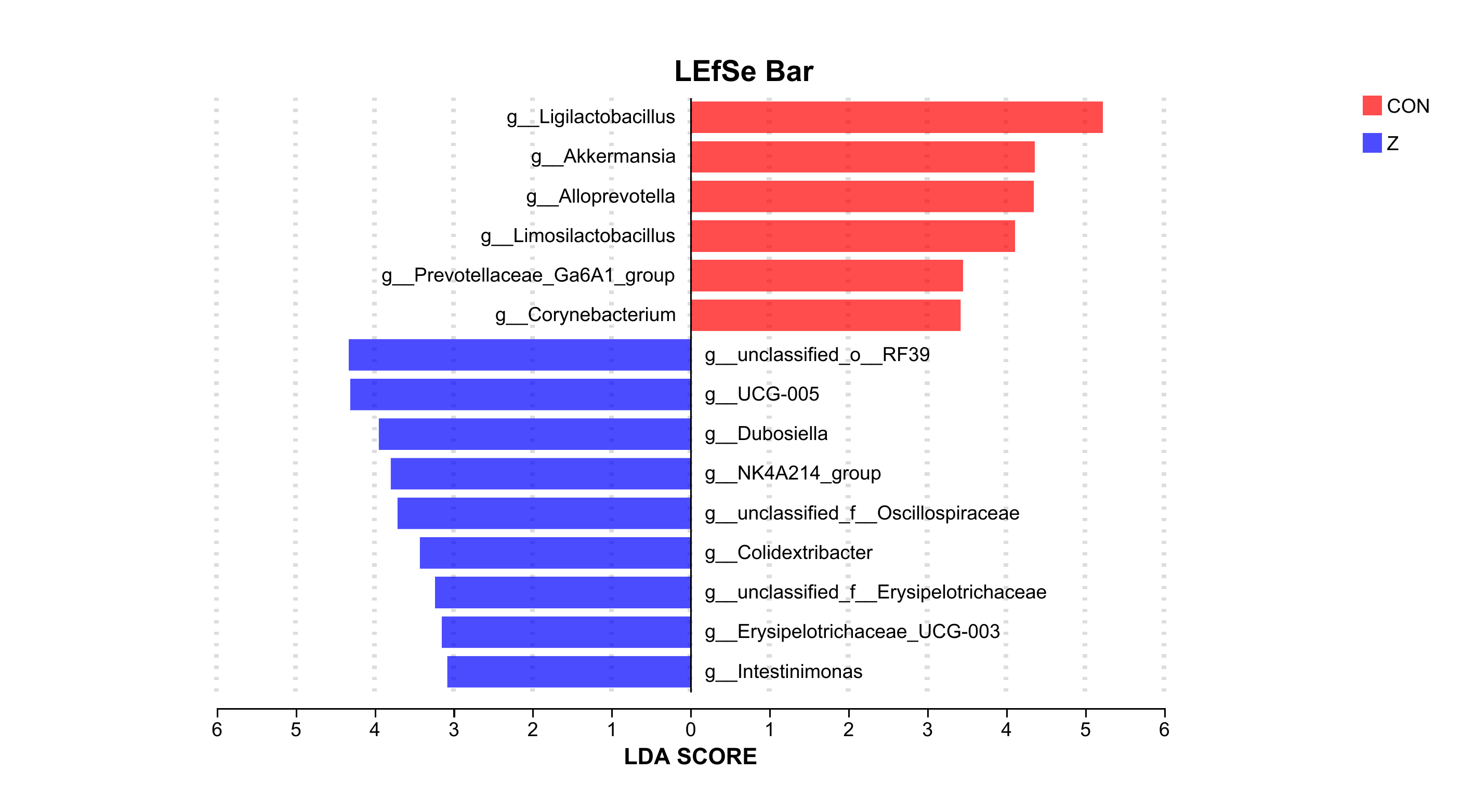


Figure S3: Genus differences in PZA alone compared to control


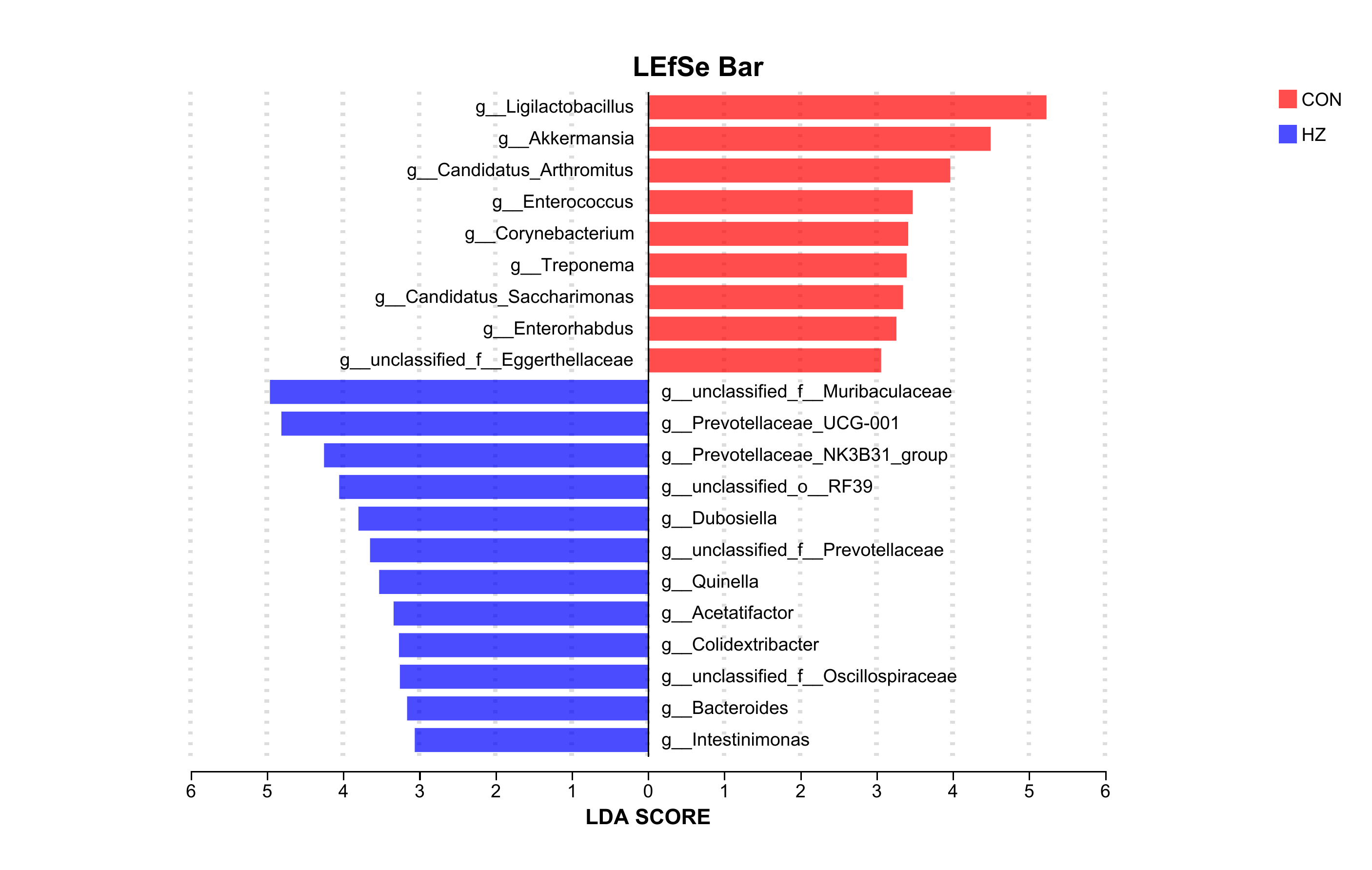


Figure S4: Genus differences in INH+PZA alone compared to control


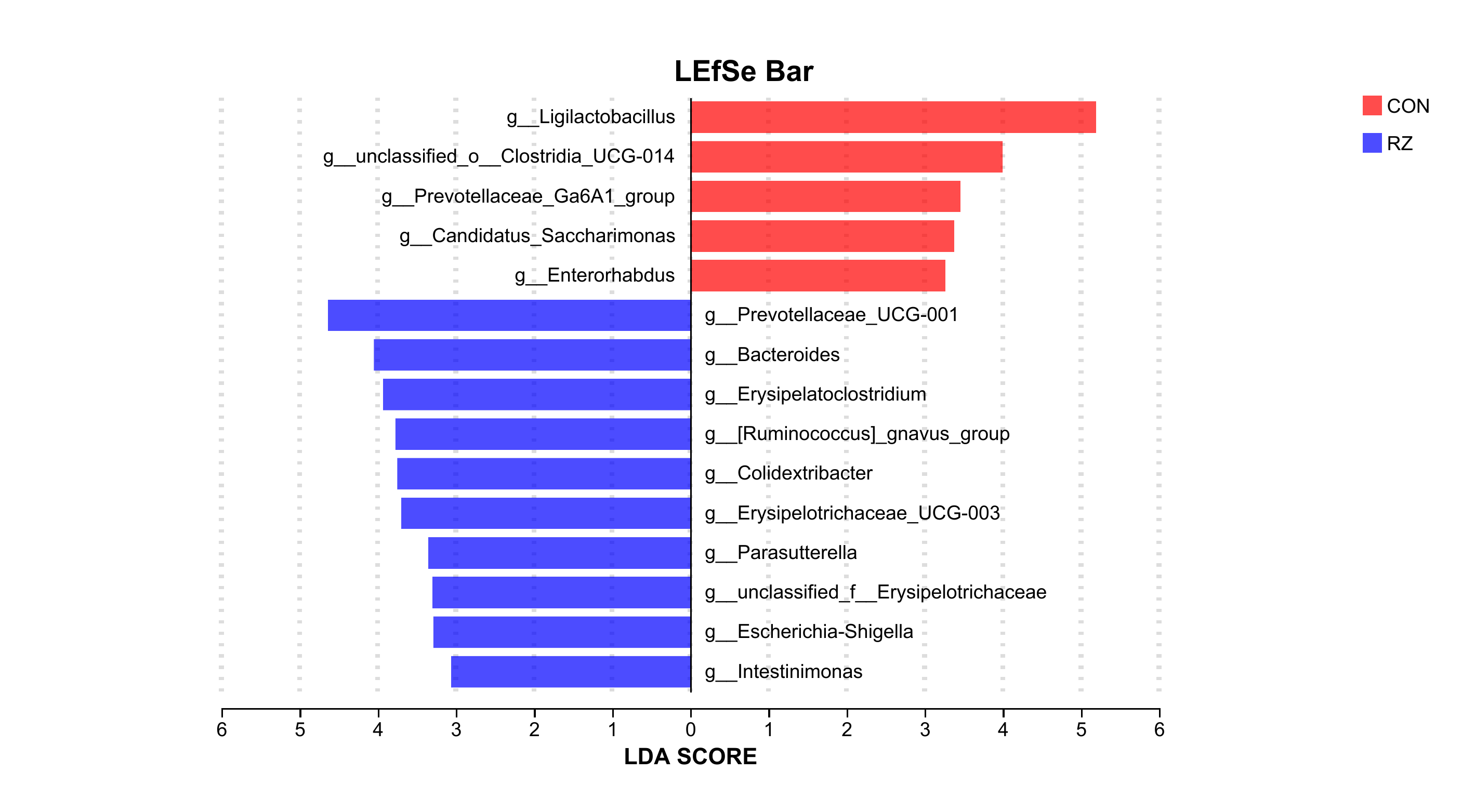


Figure S5: Genus differences in RIF+PZA alone compared to control


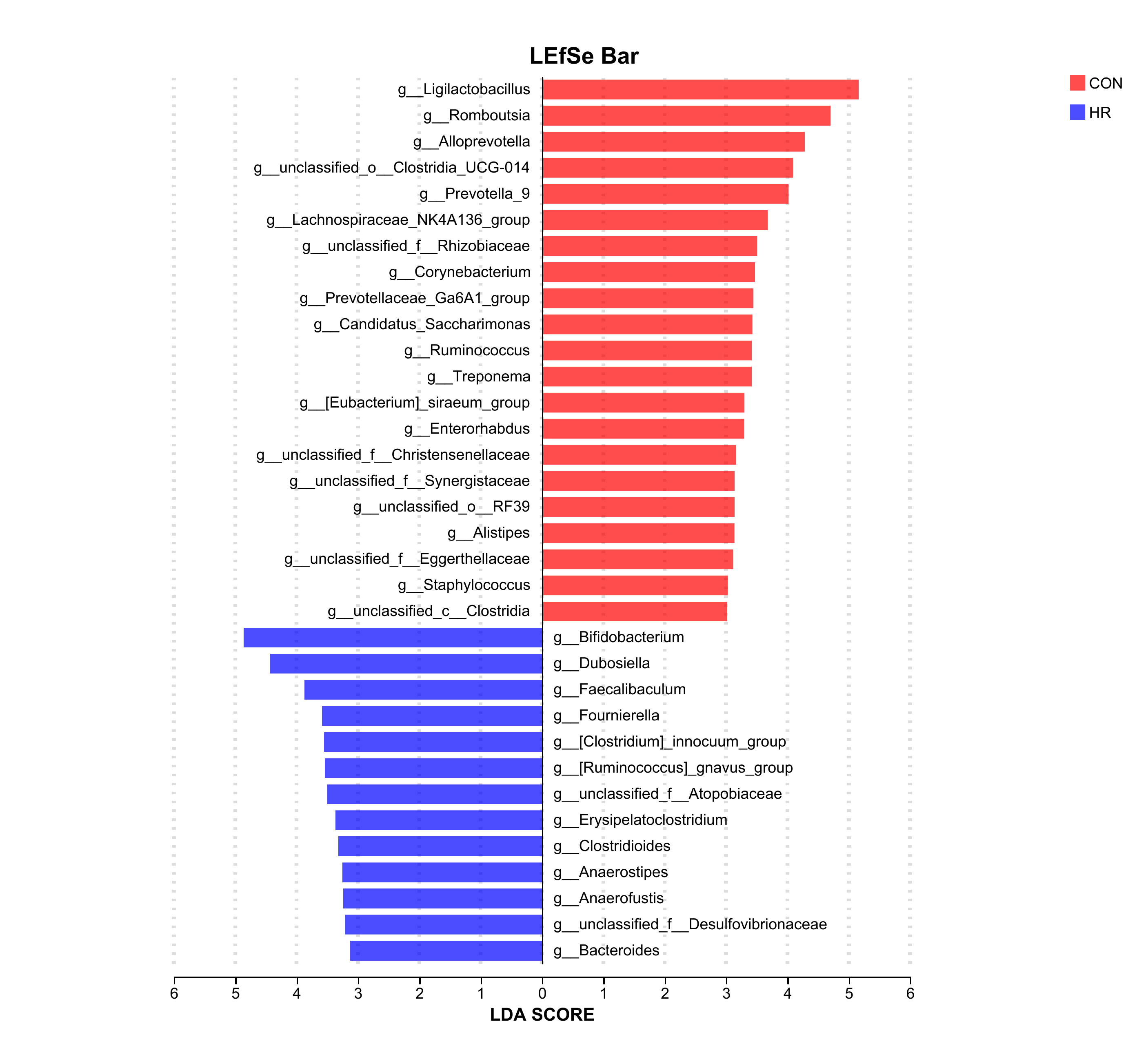


Figure S6: Genus differences in INH+RIF alone compared to control


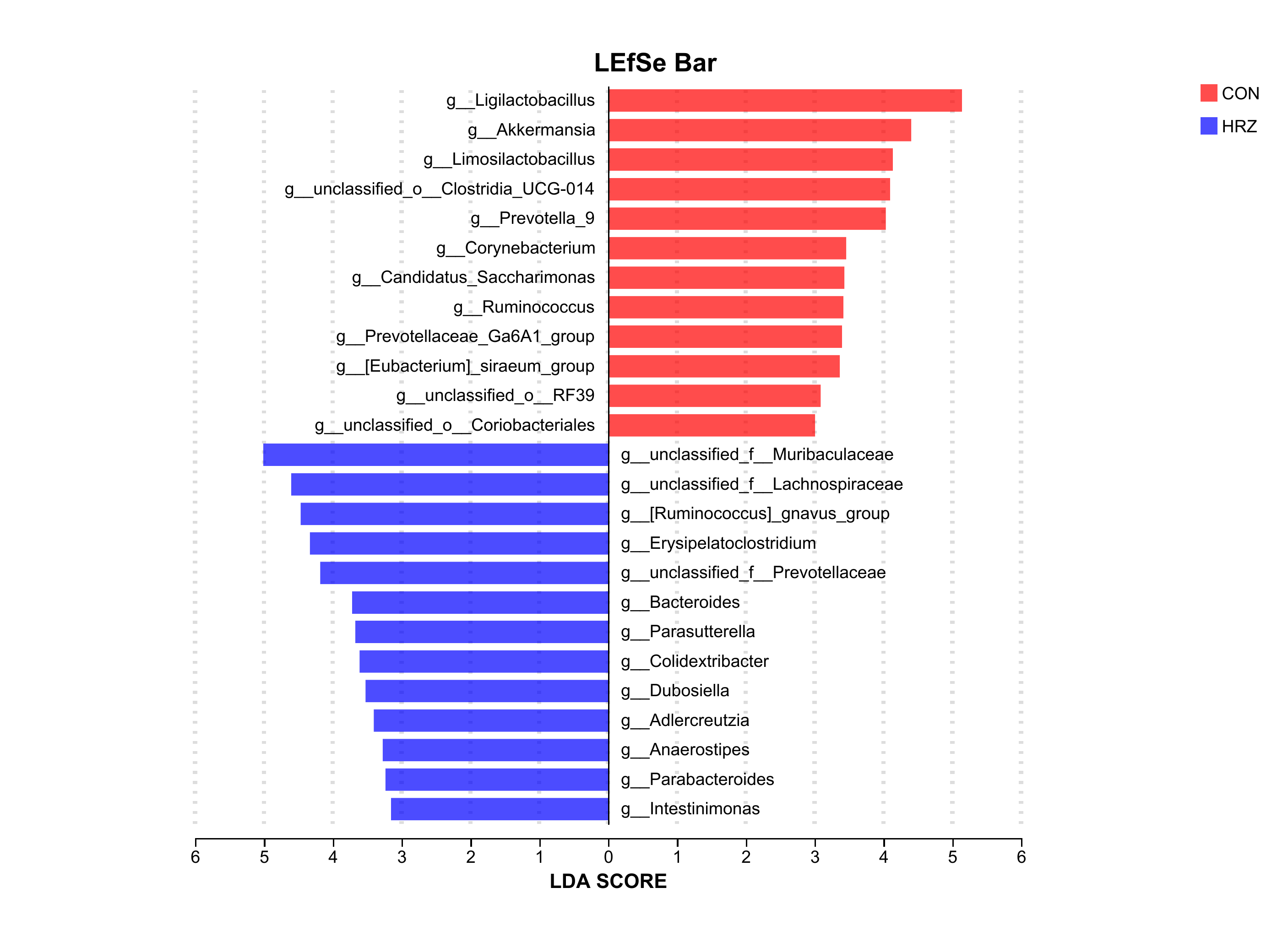


Figure S7: Genus differences in INH+RIF+PZA alone compared to control
